# Rapid and Simple Species Identification of Cicada Exuviae Using COI-Based SCAR Assay

**DOI:** 10.3390/insects11030168

**Published:** 2020-03-06

**Authors:** Pureum Noh, Wook Jin Kim, Jun-Ho Song, Inkyu Park, Goya Choi, Byeong Cheol Moon

**Affiliations:** Herbal Medicine Resources Research Center, Korea Institute of Oriental Medicine, Naju 58245, Korea; pureum322@kiom.re.kr (P.N.); ukgene@kiom.re.kr (W.J.K.); songjh@kiom.re.kr (J.-H.S.); pik6885@kiom.re.kr (I.P.); serparas@kiom.re.kr (G.C.)

**Keywords:** cicadidae periostracum, *Cryptotympana atrata*, cytochrome C oxidase subunit I, molecular authentication, sequence-characterized amplified region

## Abstract

Cicadidae periostracum (CP), the medicinal name of cicada exuviae, is well-known insect-derived traditional medicine with various pharmacological effects, e.g., anticonvulsive, anti-inflammatory, antitussive, and anticancer effects; it is also beneficial for the treatment of Parkinson’s disease. For appropriate CP application, accurate species identification is essential. The Korean pharmacopoeia and the pharmacopoeia of the People’s Republic of China define *Cryptotympana atrata* as the only authentic source of CP. Species identification of commercially distributed CP based on morphological features, however, is difficult because of the combined packaging of many cicada exuviae in markets, damage during distribution, and processing into powder form. DNA-based molecular markers are an excellent alternative to morphological detection. In this study, the mitochondrial cytochrome c oxidase subunit I sequences of *C. atrata*, *Meimuna opalifera*, *Platypleura kaempferi*, and *Hyalessa maculaticollis* were analyzed. On the basis of sequence alignments, we developed sequence-characterized amplified-region (SCAR) markers for efficient species identification. These markers successfully discriminated *C. atrata* from the three other cicada species, and detected the adulteration of market CP samples. This SCAR assay is a rapid, simple, cheap, reliable, and reproducible method for species identification, regardless of sample form and status, and contributes to CP quality control.

## 1. Introduction

Natural products such as plants, animals, micro-organisms, and even rocks have been used since prehistoric times to treat human diseases [1,2]. Insects, the largest group of living organisms, have long been used as remedies for illnesses in many regions of the world [3,4]. Various insect components, such as their bodies, eggs, egg shells, cocoons, exuviae, secretions, and toxins, have been used in traditional medicine [3]. The nymphal exuviae of the cicada are common insect-derived medicine; they are included in the ancient Korean medical book *Dongui Bogam* by Jun Heo [5]. In the Korean Herbal Pharmacopoeia, the exuviae of *Cryptotympana dubia* (Haupt, 1917) or *Cryptotympana pustulata* (Fabricius, 1787) are referred to as cicadidae periostracum (CP; sun-tae in Korean and chantui in Chinese) [6,7]. *C. dubia* and *C. pustulata* are synonyms of *Cryptotympana atrata* (Fabricius, 1775) (Cryptotympanini tribe Handlirsch), the only authentic source of CP [8,9]. In the Pharmacopoeia of the People’s Republic of China and the Taiwan Herbal Pharmacopoeia, CP is likewise specified as the exuviae of *C. atrata* [7,10,11]. 

CP has long been used as a traditional medicine to treat epilepsy, shock, smallpox, sedation, edema, and night-terror symptoms in Korea [12], and throat soreness, hoarseness, itching, and spasms in China [13]. Over the past few decades, many studies have demonstrated the pharmacological effects of CP, such as anticonvulsive, sedative, and hypothermic effects [14], as well as antianaphylactic [15], antiacne [16], antibacterial [17], anti-inflammatory [18,19], antiallergic [20], antitussive, expectorant, and antiasthmatic effects [21], and antiproliferative effects against cancer [22]. Furthermore, a recent study revealed that CP benefits patients with Parkinson’s disease by protecting dopaminergic neurons [12]. To ensure efficacy, the precise identification of the cicada species is necessary. Comparative morphological studies of cicada exuviae based on profemur length, femoral-tooth angle, distance between the intermediate and last tooth of the femoral comb, and antennae are useful for species identification [23,24,25]. However, morphological analyses are only possible when the exuviae are well-preserved, without detachment of the forelegs and antennae. In oriental-medicine markets, cicada exuviae are often packaged together, and the majority are broken into pieces during distribution. Some manufacturers process exuviae into a fine powder for convenient distribution and secondary processing. Hence, it is difficult to identify species in commercially distributed CP by morphological methods.

*Meimuna opalifera* (Walker, 1850; Dundubiini Distant tribe) and *Platypleura kaempferi* (Fabricius, 1794; Platypleurini Schmidt tribe) are common cicada species in Korea, in addition to *C. atrata* and *Hyalessa maculaticollis* (Motschulsky, 1866; Sonatini Lee tribe), the two most widespread species in Korea [26,27]. The exuviae of these four species, which share the same habitat, can be found in the same areas [26]. This can result in errors in CP collection.

Sequence-characterized amplified-region (SCAR) marker assays are PCR-based assays using 15–30 bp primers with nucleotide-sequence specificity; they enable rapid, simple, cheap, reliable, and reproducible species identification [28,29]. SCAR marker assays can be used to distinguish closely related samples by amplifying only target samples using specific primers under high annealing temperatures [28,30,31,32]. Samples can be differentiated on the basis of amplification results and length polymorphisms [28,30,31]. In animal taxa, mitochondrial DNA is commonly used for species identification, and the cytochrome c oxidase subunit I (COI) gene serves as a primary barcode region [33,34]. A previous study by Song et al. [23] verified that the COI gene is a reasonable barcode region for the discrimination of the four cicada species described above. The aims of this study were to (1) design species-specific SCAR markers for the effective discrimination of the four cicada species, *C. atrata*, *M. opalifera*, *P. kaempferi*, and *H. maculaticollis,* based on COI sequences, and (2) establish the effectiveness and sensitivity of newly developed SCAR markers by applying a SCAR assay to market CP samples.

## 2. Materials and Methods 

### 2.1. Samples and DNA Extraction

In total, 11 samples were collected for DNA barcode analysis and SCAR marker assay (Table 1). The imago bodies of *C. atrata* and *H. maculaticollis* were collected from urban areas in the summer of 2018, and the species was first identified by an expert of insect taxonomy, Bong-kyu Byun, a professor at Hannam University. Specimens of *M. opalifera* and *P. kaempferi* were provided by the Korea National Arboretum. The scientific names of the cicada species complied with Marshall et al. [35] and Liu et al. [36]. All samples were stored in 100% ethanol at −20 °C prior to genomic DNA extraction. 

Genomic DNA from one leg of each adult was extracted using the DNeasy^®^ Blood and Tissue Kit (Qiagen, Valencia, CA, USA) according to the manufacturer’s instructions. The concentration and purity of the extracted DNA were measured using a spectrophotometer (Nanodrop ND-1000; Nanodrop, Wilmington, DE, USA). Each DNA sample was adjusted to approximately 15 ng/µL prior to PCR and stored at −20 °C until further use.

### 2.2. PCR Amplification and Sequencing

The COI region was amplified using primers CO1-C02 (5′-AYTCAACAAATCATAAAG ATATTGG-3′) and CO1-C04 (5′-ACYTCRGGRTGACCAAAAAATCA-3′), developed by Che et al. [37]. Amplification was conducted in a 40 µL PCR mixture containing 0.5 μmol L^—1^ of each primer, Sol 2 × Taq PCR Smart Premix 1 (Solgent, Daejeon, Korea), and approximately 15 ng template DNA using the Pro Flex PCR System (Applied Biosystems, Life Technologies, Foster City, CA, USA). PCR thermal-cycling conditions were as follows: 95 °C for 5 min; 35 cycles of 1 min at 95 °C, 1 min at 45 °C, and 1 min at 72 °C; and a final extension for 5 min at 72 °C. PCR products were separated on a 1.5% agarose gel stained with Ecodye™ Nucleic Acid Staining Solution (Biofact, Daejeon, Korea) and visualized under UV light. The size of the PCR products was estimated using a 100 bp DNA ladder (Solgent). 

PCR products were extracted from the agarose gel using the QIAquick Gel Extraction Kit (Qiagen) and subcloned into the pGEM-T Easy Vector (Promega, Madison, WI, USA). Subcloned DNA fragments were sequenced in both directions using T7 and SP6 primers by the Sanger method. 

### 2.3. Nucleotide Sequence and Phylogenetic Analyses

The taxon assignments for COI sequences were confirmed using the NCBI BLAST search tool. The obtained sequences were deposited in the GenBank database (accession numbers are provided in Table 1). For statistical and phylogenetic analyses, 14 additional sequences, available in NCBI GenBank, were included. All sequences were aligned using ClustalW and edited using BioEdit ver. 7.2.5 [38]. Inter- and intraspecies genetic distances were calculated using MEGA ver. 6.0, following the Kimura-2-parameter (K2P) model with 1000 bootstrap replicates [39]. A phylogenetic tree was constructed using the NJ method in MEGA with 1000 bootstrap replicates on the basis of the K2P model, with pairwise deletion for gaps/missing data. The COI sequences of *Anthocharis cardamines* (MH420365) and *A. euphenoides* (GU676624) served as an outgroup in phylogenetic analysis.

### 2.4. SCAR Marker Development and Market-Sample-Authenticity Test

To avoid errors arising from sampling bias, the four following sequences were included for SCAR marker development: *C. atrata*, MG737717; *M. opalifera*, GQ527088; *P. kaempferi*, MG737816; and *H. maculaticollis*, KY860344. A total of 15 nucleotide sequences were aligned using ClustalW and edited using BioEdit [38]. On the basis of the aligned sequences, we searched several short regions (19–23 bp) that contained at least two species-specific nucleotides, and tried to design primers to include species-specific nucleotides close to the 3’ end. PCRs were performed to investigate the effectiveness of each primer combination, and the best primer set generating consistent results was selected. Amplification was conducted in a 20 µL PCR mixture containing 0.5 μmol L^−1^ of each species-specific forward and reverse primer (Table 2), Solg 2 × Taq PCR Smart Premix 1 (Solgent), and approximately 15 ng template DNA using the Pro Flex PCR System (Applied Biosystems, Life Technologies). The following thermal cycling conditions were used: 95 °C for 2 min; 35 cycles of 20 s at 95 °C, 30 s at 55 °C, and 20 s at 72 °C; and a final extension for 5 min at 72 °C. PCR products were visualized by gel electrophoresis on 1.5% agarose gel, as described in Section 2.2.

The sensitivity of the developed primers was evaluated using five different genomic DNA concentrations (1.5 ng, 150 pg, 15 pg, 1.5 pg, and 150 fg). Additional PCRs were performed using a template DNA that was serially diluted 10-fold with an final elution buffer, AE, with the same PCR and gel electrophoresis conditions described above.

CP samples originating in China were purchased from oriental-medicine markets in Korea (n = 7) and China (n = 1) (Table S1). To detect whether the samples were adulterated, at least three exuviae with shape differences observable with the naked eye in each package were selected and ground (Figure S1). Genomic DNA was extracted from the ground samples according to the protocol described above, and PCR was performed using the primer set selected by the SCAR marker assay. 

## 3. Results

### 3.1. Nucleotide Sequence and Phylogenetic Analyses

A total of 25 COI sequences (628 bp), including sequences from 11 cicada samples and the GenBank database, were used for statistical and phylogenetic analyses. Intra- and inter-species distances ranged from 0.016 ± 0.0010 to 0.0027 ± 0.0037, and 0.2020 ± 0.0183 to 0.2320 ± 0.0112, respectively (Table S2). A phylogenetic tree was inferred using the COI sequences of the four cicada species on the basis of the neighbor-joining method (Figure S2). The phylogenetic tree showed that each species clustered into a monophyletic group with high bootstrap support (99%–100%; Figure S2). This result demonstrated that COI region sequences could be used to discriminate the four cicada species. According to Figure S2, *H. maculaticollis* and *M. opalifera* samples were more closely related than the other samples. 

### 3.2. Development of Species-Specific SCAR Markers

**Figure 1:**
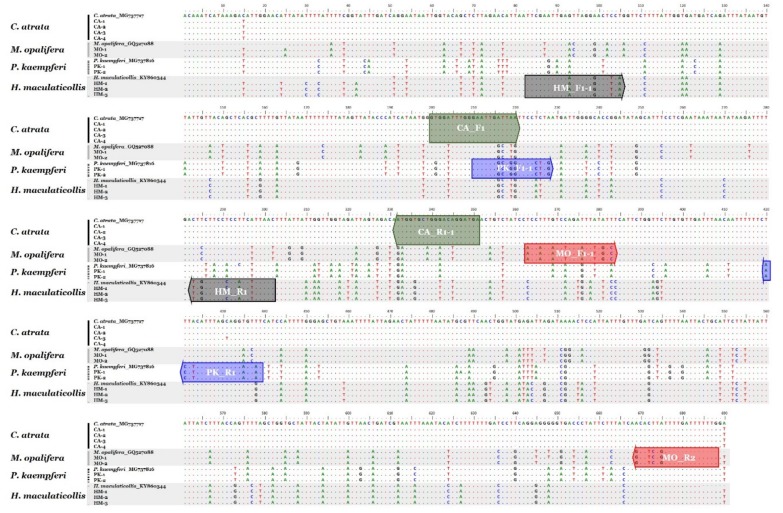
Comparative sequence analysis of cytochrome c oxidase subunit I (COI) region in four cicada species. Positions and directions of four species-specific primer pairs (box-shaped arrows) displayed on sequences.

**Figure 2:**
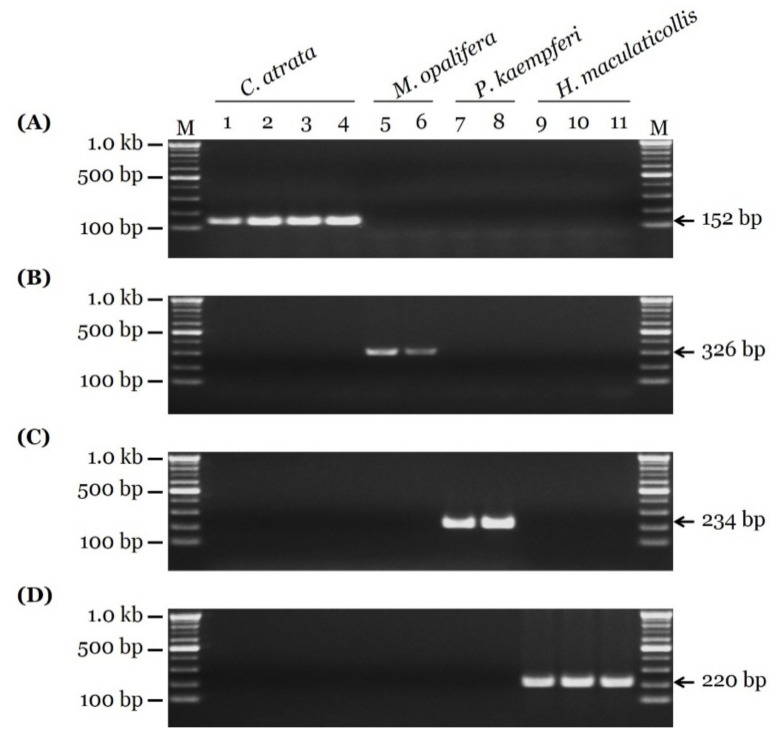
Gel images showing species-specific SCAR PCR results. Fragments of COI regions of (**A**) *C. atrata*, (**B**) *M. opalifera*, (**C**) *P. kaempferi*, and (**D**) *H. maculaticollis* were produced using CA_F1/CA_R1-1, MO_F1-1/MO_R2, PK_F1-1/PK_R1, and HM_F1-1/HM_R1 primer pairs, respectively. Primer sequences listed in Table 2. Lanes 1 to 4 correspond to Samples CA-1 to CA-4, respectively, of *C. atrata*; Lanes 5 and 6 correspond to Samples MO-1 and MO-2, respectively, of *M. opalifera*; Lanes 7 and 8 correspond to Samples PK-1 and PK-2, respectively, of *P. kaempferi*; Lanes 9 to 11 correspond to Samples HM-1 to HM-3, respectively, of *H. maculaticollis*. Information of 11 samples provided in Table 1. Lane M represents 100 bp DNA ladder. Arrows indicate precise sizes of PCR products.

From the 11 cicada samples, 690 bp of COI sequences were obtained. In addition, four sequences from the GenBank database were included, for a total of 15 sequences. On the basis of comparative sequence analysis of the COI sequences, nucleotide polymorphisms for species identification were detected (Figure 1). On the basis of these nucleotide polymorphisms, species-specific primer pairs were developed and optimized (Figure 1 and Table 2). These primer pairs produced PCR products of 152, 326, 234, and 220 bp for *C. atrata*, *M. opalifera*, *P. kaempferi*, and *H. maculaticollis*, respectively (Figure 2). PCR product sizes were proven to be unique and discriminable by comparing the bands on agarose gel (Figure S3).

### 3.3. Sensitivity Test and Authenticity of Market Samples

**Figure 3:**
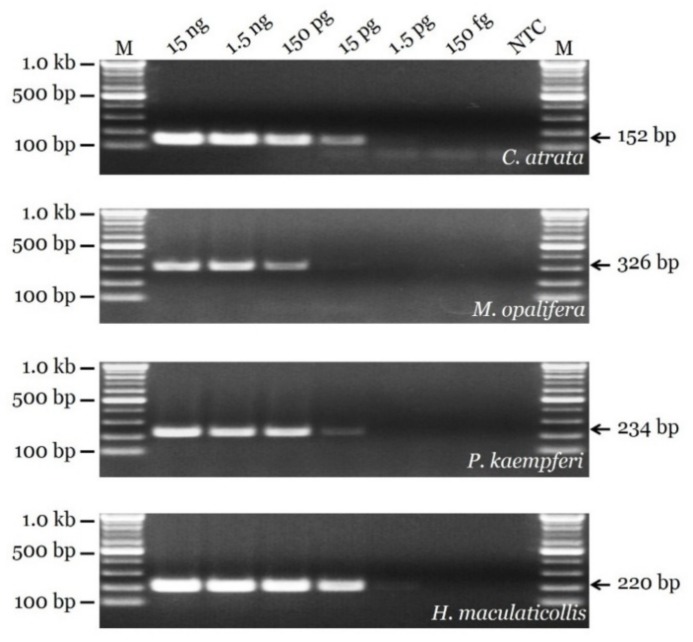
PCR sensitivity of newly developed SCAR markers evaluated using serially diluted template DNA. Lane M represents 100 bp DNA ladder. Arrows indicate precise sizes of PCR products.

The results of a PCR sensitivity test using serially diluted template DNA are presented in Figure 3. Four primer pairs were effective for templates of 15 ng, 1.5 ng, and 150 pg. A significant difference in sensitivity between the primers was detected in the 15 pg condition. The strongest band was obtained for the HM_F1-1/HM_R1 pair, and the weakest band was obtained for the MO_F1-1/MO_R2 pair. For 1.5 pg, only the HM_F1-1/HM_R1 primer pair generated a visible band. No primer pairs generated a visible PCR product for 150 fg.

**Figure 4:**
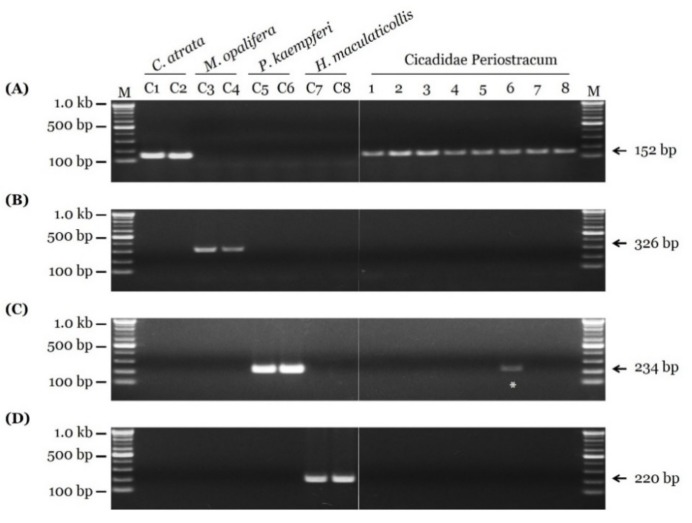
Evaluation of eight market samples using newly developed SCAR markers. Fragments of COI regions of (**A**) *C. atrata*, (**B**) *M. opalifera*, (**C**) *P. kaempferi*, and (**D**) *H. maculaticollis* were produced using CA_F1/CA_R1-1, MO_F1-1/MO_R2, PK_F1-1/PK_R1, and HM_F1-1/HM_R1 primer pairs, respectively. Gel images of (left) PCR products of control samples (C1: CA-1, C2: CA-2, C3: MO-1, C4: MO-2, C5: PK-1, C6: PK-2, C7: HM-1, and C8: HM-2) and (right) market CP samples. Asterisk, adulterated sample. Details of market samples presented in Table S1.

Eight market samples were examined using species-specific SCAR markers (Figure 4). Only *C. atrata,* indicating authentic CP, was detected in the market samples, with the exception of Sample 6, which contained both *C. atrata*, and *P. kaempferi* (Figure 4 and Figure S4). Therefore, the developed SCAR markers in this study are useful to discriminate between adulterated and unadulterated samples, and confirmed that adulterated CP products are currently distributed.

## 4. Discussion

The use of natural products as medicine requires precise discrimination between medicinal sources from other taxa [1]. In the past, natural products were identified by experts on the basis of appearance, smell, or taste [40]. This method has many limitations, including the risk of human error, difficulty of acquiring the appropriate skills, and variation in the natural populations of organisms. Moreover, medicinal natural products are sold in processed forms, such as powder, sections, and globules; thus, morphological identification is almost impossible [41]. Recently, microscopes have been used to identify medicinal sources. A previous study by Song et al. [23] suggested several morphological characteristics of forelegs and ultrastructures that help distinguish *C. atrata*, *M. opalifera*, *P. kaempferi,* and *H. maculaticollis*. This morphological method requires well-preserved cicada exuviae without damage to the original form. Therefore, morphological discrimination is impracticable to broken and processed CP. A more reliable and objective method for the identification of authentic CP that is applicable to any processed form was needed for medicinal use of CP.

Technological advances enabled DNA-based authentication that is highly reliable. DNA barcoding (i.e., identification based on species-specific variants of short DNA fragments) is an effective and accurate method, and it is now generally accepted for taxon diagnosis [33,42]. Bouwer et al. [43] reported the molecular identification of a cicada species, *Quintilia carinata* (Thunberg, 1822), using DNA sequence data from its exuviae. However, nucleotide-sequencing and -analysis procedures can be expensive and time consuming. SCAR markers simplify the process into three steps, namely, DNA extraction, PCR amplification, and gel electrophoresis [40,44].

We developed SCAR markers and established a SCAR assay for the discrimination of four cicada species. The SCAR markers successfully discriminated between *C. atrata*, the authentic source species of CP, and three other cicada species, even at low DNA concentrations. Length differences in the PCR products produced by species-specific SCAR primers enabled the identification of species based on comparisons of band sizes by gel electrophoresis. Accordingly, this is a rapid, simple, cheap, reliable, and reproducible method to investigate the adulteration of commercially distributed CP, regardless of its shape and form.

China is a major producer and exporter of traditional herbal medicines; traditional Chinese herbal medicines are exported to about 163 countries [45]. Korea is a major importer of traditional Chinese herbal medicines [46]. In China, *Auritibicen flammatus* (Distant, 1892), *Cryptotympana mandarina* (Distant, 1891) (both Cryptotympanini Handlirsch tribe), and *P. kaempferi* are also used to produce CP because of the high demand for and decline of *C. atrata* populations [47]. There is a strong possibility that inauthentic or adulterated CP is distributed around the world. We found clear evidence for adulteration. The newly developed SCAR markers detected *P. kaempferi,* which is not an authentic CP source, in a market CP sample (Figure 4 and Figure S4, and Table S1). Therefore, further studies are needed to develop SCAR markers for Chinese cicada species aimed at global consumers, including Korean consumers.

## 5. Conclusions

We developed SCAR markers to discriminate between *C. atrata* and three other cicada species for the accurate identification and application of CP. The primer pairs designed to target each species were effective even at low DNA concentrations, and enabled species identification by comparison of PCR product sizes. Our results provide a rapid, simple, cheap, reliable, and reproducible method to investigate the adulteration of commercially distributed CP, regardless of form and status.

## Figures and Tables

**Table 1:** Sources of cicada species used for sequence analyses and marker development.

Species	Collection Site	Collection Date	Voucher Number	Abbrev.	GenBank Accession
*Cryptotympana atrata*	Yuseong-gu, Daejeon, Korea	16 August 2018	2-19-0006	CA-1	MN784082
	Yuseong-gu, Daejeon, Korea	16 August 2018	MBC-KIOM-2018-12	CA-2	MN784083
	Yuseong-gu, Daejeon, Korea	20 August 2018	MBC-KIOM-2018-14	CA-3	MN784084
	Yuseong-gu, Daejeon, Korea	20 August 2018	MBC-KIOM-2018-20	CA-4	MN784085
*Meimuna opalifera*	Idong-myeon, Namhae-gun, Gyeongsangnam-do, Korea	26 August 2014	KNAE453746-no.16	MO-1	MN784086
	Aphae-eup, Sinan-gun, Jeollanam-do, Korea	29 August 2011	KNAE301381-no.25	MO-2	MN784087
*Platypleura kaempferi*	Gyodong-myeon, Ganghwa-gun, Incheon, Korea	7 July 1998	KNAE29864-no.19	PK-1	MN784088
	Soheul-eup, Pocheon-si, Gyeonggi-do, Korea	3 July 2002	KNAE43938-no.21	PK-2	MN784089
*Hyalessa maculaticollis*	Gwangmyeong-si, Gyeonggi-do, Korea	27 July 2018	2-19-0007	HM-1	MN784090
	Yuseong-gu, Daejeon, Korea	28 July 2018	MBC-KIOM-2018-16	HM-2	MN784091
	Yeongdeungpo-gu, Seoul, Korea	29 July 2018	MBC-KIOM-2018-18	HM-3	MN784092

**Table 2:** Sequences of sequence-characterized amplified-region (SCAR) primers and product sizes.

Target Species	Primer Name ^1^	Primer Sequence (5′→3′)	PCR Product Size (bp)
*Cryptotympana atrata*	CA_F1	GTGGATTTGGGAATTGATTAA	152 bp
	CA_R1-1	TCATCCTGTCCCAGCACCAT	
*Meimuna opalifera*	MO_F1-1	ACCATTATCTAGAATTTTGTC	326 bp
	MO_R2	CAAAAAATCAAAACAGATGC	
*Platypleura kaempferi*	PK_F1-1	GAATTGGCTGGTTCCCTTG	234 bp
	PK_R1	ATACTCCTGCTAAATGAAGT	
*Hyalessa maculaticollis*	HM_F1-1	TTCGAATTGAATTAGGGACTTCA	220 bp
	HM_R1	GTTAAAGATGGGGGAAGCAA	

^1^ F and R, forward and reverse primers, respectively.

## Supplementary Materials

The following are available online at https://www.mdpi.com/2075-4450/11/3/168/s1. Figure S1: Diagram of sample preparation for market-sample-authenticity test. Figure S2: Neighbor-joining tree based on COI sequences of four cicada species, with 1000 bootstrap replicates. Figure S3: Gel image comparing sizes of species-specific SCAR PCR products. Figure S4: Picture of market-sample 6. Table S1. List of cicadidae periostracum samples purchased from oriental-medicine markets for verification of newly developed SCAR marker assay. Table S2: Statistical characteristics of COI sequences.

## References

[1] Yuan H., Ma Q., Ye L., Piao G. (2016). The Traditional Medicine and Modern Medicine from Natural Products. Molecules.

[2] Heo J. (2009). Dongui-Bogam: Treasured Mirror of Eastern Medicine.

[3] Feng Y., Zhao M., He Z., Chen Z., Sun L. (2009). Research and utilization of medicinal insects in China. Entomol. Res..

[4] Srivastava S.K., Babu N., Pandey H. (2009). Traditional insect bioprospecting—As human food and medicine. Indian J. Tradit. Knowl..

[5] Song B.-K., Won J.-H., Kim S. (2016). Historical medical value of Donguibogam. J. Pharmacopunct..

[6] Ministry of Food and Drug Safety (2013). The Korean Herbal Pharmacopoeia.

[7] Defining Dictionary for Medicinal Herbs [Korean, ‘Hanyak Giwon Sajeon’]. http://boncho.kiom.re.kr/codex/.

[8] Park J., Choi G. (2016). Review on herbal medicinal materials in the Korean Pharmacopoeia and the Korean Herbal Pharmacopoeia. Korean Herb. Med. Inf..

[9] Song J.-H., Yang S., Kim W.J., Moon B.C. (2019). Studies on morphological characteristics of the original species, *Cryptotympana atrata* as a Cicadidae Periostracum. Korean Herb. Med. Inf..

[10] Chinese Pharmacopoeia Commission (2015). Pharmacopoeia of the People’s Republic of China.

[11] Committee on Chinese Medicine and Pharmacy (2013). Taiwan Herbal Pharmacopoeia.

[12] Lim H.-S., Kim J.-S., Moon B.C., Choi G., Ryu S.M., Lee J., Ang M.J., Jeon M., Moon C., Park G. (2019). Cicadidae Periostracum, the Cast-Off Skin of Cicada, Protects Dopaminergic Neurons in a Model of Parkinson’s Disease. Oxid. Med. Cell. Longev..

[13] Pui-hay B.P., Hson-mou C. (1986). Pharmacology and Applications of Chinese Materia Medica.

[14] Hsieh M.T., Peng W.H., Yeh F.T., Tsai H.Y., Chang Y.S. (1991). Studies on the anticonvulsive, sedative and hypothermic effects of Periostracum Cicadae extracts. J. Ethnopharmacol..

[15] Shin T.Y., Park J.H., Kim H.M. (1999). Effect of *Cryptotympana atrata* extract on compound 48/80-induced anaphylactic reactions. J. Ethnopharmacol..

[16] Lim J.-P. (2010). Acne-remedy effects of extract mixture of Pulsatillae Radix and Cicadidae Periostracum. J. Physiol. Pathol. Korean Med..

[17] Wang J., Tian Q., Tao G., Gao Q., Lv T., Wang D. (2010). Analyses on ingredients and antibacterial activity of periostracum cicadae. Chin. Bull. Entomol..

[18] Kim K.-W., Cho H.-B., Kim S.-B., Choe C.-M., Seo Y.-J. (2011). The anti-inflammatory effects of Cicadidae Periostracum. J. Orient. Obstet. Gynecol..

[19] Xu M.-Z., Lee W.S., Han J.-M., Oh H.-W., Park D.-S., Tian G.-R., Jeong T.-S., Park H.-Y. (2006). Antioxidant and anti-inflammatory activities of N-acetyldopamine dimers from Periostracum Cicadae. Bioorg. Med. Chem..

[20] Kim B.N.R., Chae J.W. (2015). Effects of Cicadae Periostracum (CP) in allergic contact dermatitis (ACD) induced by DNCB in mice. J. Pediatr. Korean Med..

[21] Xu S.-N., Zhang M.-Y., Wang Y.-M., Jin Y.-R., Liu Z.-M. (2007). Antitussive, expectorant and antiasthmatic effects of periostracum cicadae. Chin. Pharmacol. Bull..

[22] Song H.E. (2014). Anti-Proliferative Activity of Constituent Identified in Cicada Slough on Human Prostate Cancer. Master’s Thesis.

[23] Song J.-H., Kim W.J., Cha J.-M., Yang S., Choi G., Moon B.C. (2019). Comparative morphological, ultrastructural, and molecular studies of four cicadinae species using exuvial legs. Insects.

[24] Lee H.-Y., Oh S.-Y., Jang Y. (2012). Morphometrics of the final instar exuviae of five cicada species occurring in urban areas of central Korea. J. Asia Pacif. Entomol..

[25] Hou Z., Li Q., Wei C. (2014). Morphology and identification of the final instar nymphs of three cicadas (Hemiptera, Cicadidae) in Guanzhong Plain, China based on comparative morphometrics. ZooKeys.

[26] Lee Y.J. (2005). Cicadas of Korea.

[27] Lee Y.J. (2008). Revised synonymic list of Cicadidae (Insecta: Hemiptera) from the Korean Peninsula, with the description of a new species and some taxonomic remarks. Proc. Biol. Soc. Wash..

[28] Kim W.J., Moon B.C., Yang S., Han K.S., Choi G., Lee A.Y. (2016). Rapid authentication of the herbal medicine plant species *Aralia continentalis* Kitag. and *Angelica biserrata* C.Q. Yuan and R.H. Shan using ITS2 sequences and multiplex-SCAR markers. Molecules.

[29] Noh P., Kim W.J., Yang S., Park I., Moon B.C. (2018). Authentication of the herbal medicine Angelicae Dahuricae Radix using an ITS sequence-based multiplex SCAR assay. Molecules.

[30] Moon B.C., Kim W.J., Han K.S., Yang S., Kang Y., Park I., Piao R. (2017). Differentiating authentic Adenophorae Radix from its adulterants in commercially-processed samples using multiplexed ITS sequence-based SCAR markers. Appl. Sci..

[31] Bhagyawant S.S. (2016). RAPD-SCAR markers: An interface tool for authentication of traits. J. Biosci. Med..

[32] Cheng J., Long Y., Khan A., Wei C., Fu S., Fu J. (2015). Development and significance of RAPD-SCAR markers for the identification of *Litchi chinensis* Sonn. by improved RAPD amplification and molecular cloning. Electron. J. Biotechnol..

[33] Hebert P.D., Cywinska A., Ball S.L., Dewaard J.R. (2003). Biological identifications through DNA barcodes. Proc. R. Soc. Lond. Ser. B Biol. Sci..

[34] Syromyatnikov M.Y., Borodachev A.V., Kokina A.V., Popov V.N. (2018). A molecular method for the identification of honey bee subspecies used by beekeepers in Russia. Insects.

[35] Marshall D.C., Moulds M., Hill K.B., Price B.W., Wade E.J., Owen C.L., Goemans G., Marathe K., Sarkar V., Cooley J.R. (2018). A molecular phylogeny of the cicadas (Hemiptera: Cicadidae) with a review of tribe and subfamily classification. Zootaxa.

[36] Liu Y., Qiu Y., Wang X., Yang H., Hayashi M., Wei C. (2018). Morphological variation, genetic differentiation and phylogeography of the East Asia cicada *Hyalessa maculaticollis* (Hemiptera: Cicadidae). Syst. Entomol..

[37] Che J., Chen H.-M., Yang J.-X., Jin J.-Q., Jiang K., Yuan Z.-Y., Murphy R.W., Zhang Y.-P. (2012). Universal COI primers for DNA barcoding amphibians. Mol. Ecol. Res..

[38] Hall T.A. (1999). BioEdit: A user-friendly biological sequence alignment editor and analysis program for Windows 95/98/NT. Nucleic Acids Symp. Ser..

[39] Tamura K., Stecher G., Peterson D., Filipski A., Kumar S. (2013). MEGA6: Molecular evolutionary genetics analysis version 6.0. Mol. Biol. Evol..

[40] Shen Z., Lu T., Zhang Z., Cai C., Yang J., Tian B. (2019). Authentication of traditional Chinese medicinal herb “Gusuibu” by DNA-based molecular methods. Ind. Crops. Prod..

[41] Osathanunkul M., Osathanunkul R., Madesis P. (2018). Species identification approach for both raw materials and end products of herbal supplements from Tinospora species. BMC Complement. Altern. Med..

[42] Chen S., Pang X., Song J., Shi L., Yao H., Han J., Leon C. (2014). A renaissance in herbal medicine identification: From morphology to DNA. Biotechnol. Adv..

[43] Bouwer N., Midgley J.M., Timm A.E., Villet M.H. (2014). Successful identification of the final instar nymph of *Quintilia carinata* (Thunberg) (Hemiptera: Cicadidae) by DNA extraction from the exuvium. J. Nat. Hist..

[44] Li F.-W., Kuo L.-Y., Huang Y.-M., Chiou W.-L., Wang C.-N. (2010). Tissue-direct PCR, a rapid and extraction-free method for barcoding of ferns. Mol. Ecol. Res..

[45] Xu J., Yang Y. (2009). Traditional Chinese medicine in the Chinese health care system. Health Policy.

[46] Pemberton R.W. (1999). Insects and other arthropods used as drugs in Korean traditional medicine. J. Ethnopharmacol..

[47] Cao X.-C., Zhang X.-Y., Xu J.-D., Shen H., Zhou S.-S., Zhu H., Kong M., Zhang W., Zhou G.-R., He Y. (2019). Quality consistency evaluation on four origins of Cicadae Periostracum by ultra-performance liquid chromatography coupled with quadrupole/time-of-flight mass spectrometry analysis. J. Pharm. Biomed. Anal..

