# Supplementary material for: Rapid and Simple Species Identification of Cicada Exuviae Using COI-Based SCAR Assay
Source: Insects. 2020 Mar 6;11(3):168. doi: 10.3390/insects11030168 (PMC7143061; doi:10.3390/insects11030168)
Supplement: Supplementary file 1 [file insects-11-00168-s001.pdf]

Supplementary Materials: Rapid and Simple Species Identification of Cicada Exuviae using a COI-based SCAR Assay

Pureum Noh, Wook Jin Kim, Jun-Ho Song, Inkyu Park, Goya Choi, and Byeong Cheol Moon *

Table S1. List of Cicadidae Periostracum samples purchased from oriental medicine markets for the verification of the newly developed SCAR marker assay.

Crude Medicine Name	Manufacturer (Country)	Country of Origin	Purchase Year	Sample Number = Lane in Gel (Figure 4)
Cicadidae Periostracum	S○○ pharmaceutical company (China)	China	2015	1
	G○○ pharmaceutical company (South Korea)	China	2018	2
	G○○ pharmaceutical company (South Korea)	China	2019	3
	G○○ pharmaceutical company (South Korea)	China	2019	4
	P○○ pharmaceutical company (South Korea)	China	2019	5
	P○○ pharmaceutical company (South Korea)	China	2019	6
	J○○ pharmaceutical company (South Korea)	China	2019	7
	H○○ pharmaceutical company (South Korea)	China	2019	8

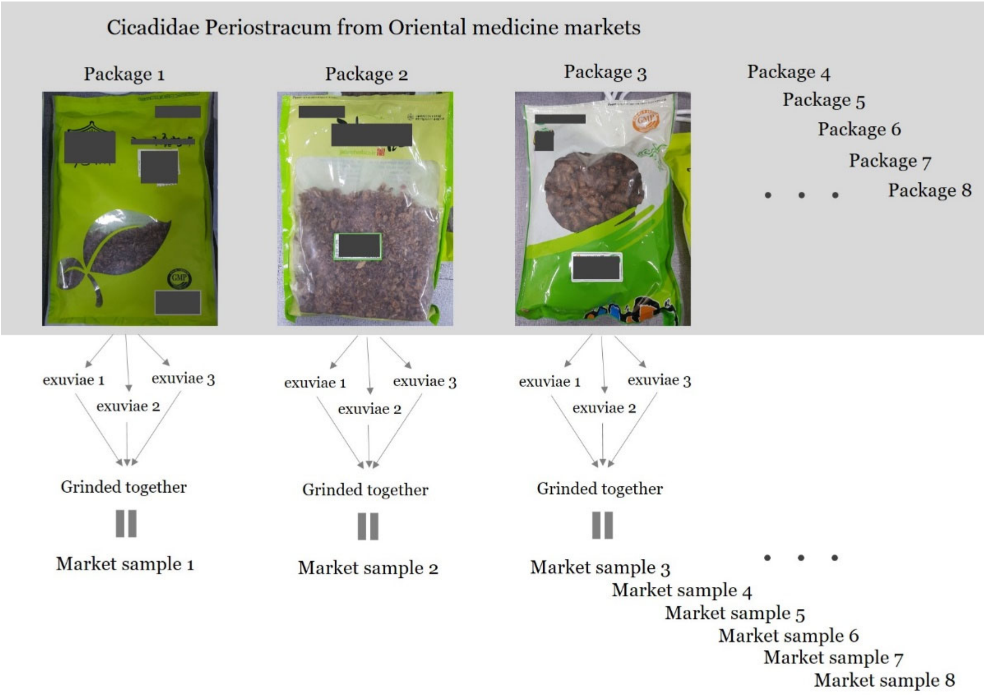

Figure S1. A diagram of sample preparation for the market sample authenticity test.

Table S2. Statistical characteristics of COI sequences

Species	Amplicon length (bp)	Intra-specific distance	Inter-specific distance	G+C (%)
Cryptotympana atrata	628	0.0024 ± 0.0035	0.2250 ± 0.0136	31.2
Meimuna opalifera	628	0.0016 ± 0.0010	0.2020 ±0.0183	31.3
Platypleura kaempferi	628	0.0019 ± 0.0014	0.2320 ± 0.0112	30.3
Hyalessa maculaticollis	628	0.0027 ± 0.0037	0.2112 ± 0.0198	31.1

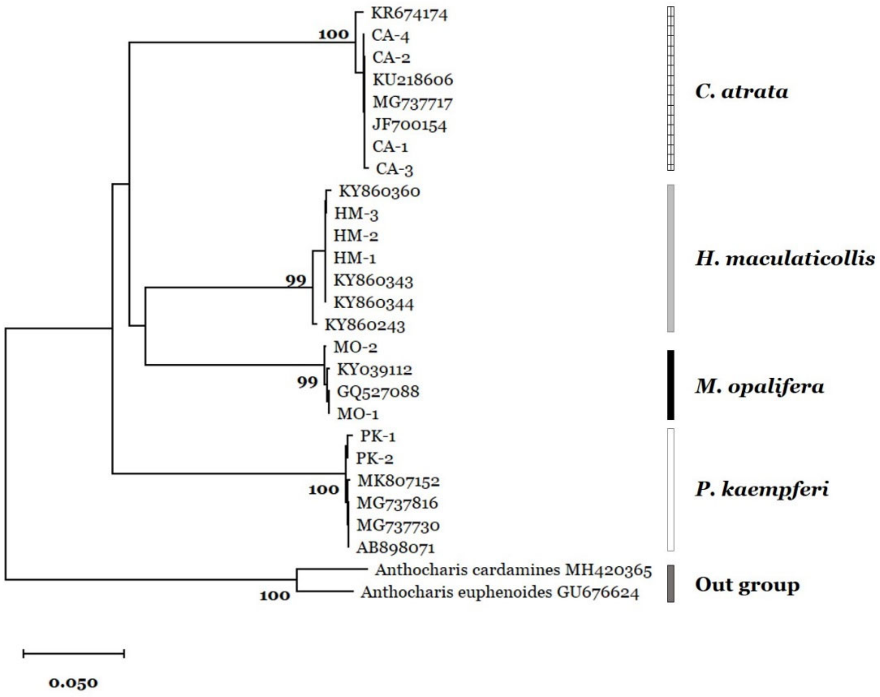

Figure S2. Neighbor-joining tree based on COI sequences of four cicada species, with 1000 bootstrap replicates. Bootstrap values are presented at the nodes. Scale bar represents 0.05 substitutions per site.

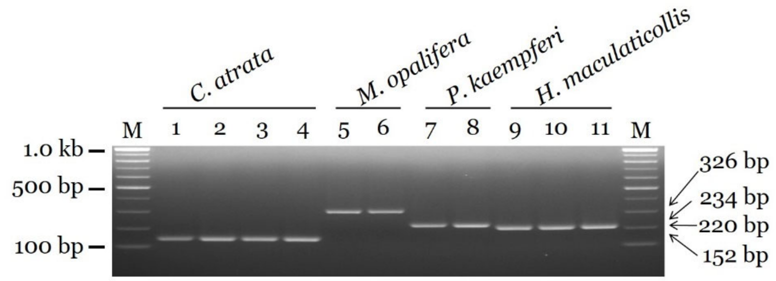

Figure S3. A gel image comparing the sizes of species-specific SCAR PCR products. Fragments of COI regions of *C. atrata*, *M. opalifera*, *P. kaempferi*, and *H. maculaticollis* were produced using CA_F1/CA_R1-1, MO_F1-1/MO_R2, PK_F1-1/PK_R1, and HM_F1-1/HM_R1 primer pairs, respectively. Lane numbers correspond to those of Figure 2. Information for the 11 samples is provided in Table 1. Lane M represents the 100 bp DNA ladder. Arrows indicate the precise sizes of PCR products.

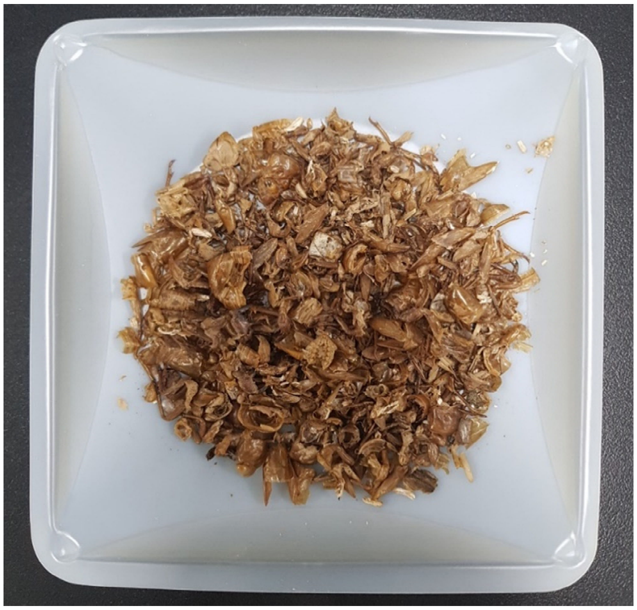

Figure S4. Picture of market sample number 6. Cicada exuviae were broken into pieces, making it impossible to identify species using morphological features. In this sample, *P. kaempferi* (not an authentic source) and *C. atrata* (an authentic source of Cicadidae Periostracum) were detected using the SCAR markers developed in this study.
